# Renal artery pseudoaneurysm following holmium laser diverticular neck incision for calyceal diverticular stones: a rare case report

**DOI:** 10.3389/fsurg.2025.1743526

**Published:** 2026-01-05

**Authors:** Botao Yu, Chunling Wang, Ningying Zhou, Min Yin

**Affiliations:** 1 Department of Urology, Ningbo Medical Center Lihuili Hospital, Ningbo, Zhejiang, China; 2 Department of Radiation Oncology, Ningbo Medical Center Lihuili Hospital, Ningbo, Zhejiang, China

**Keywords:** calyceal diverticulum, flexible ureteroscopy, hemorrhagic complication, laser lithotripsy, renal artery embolization

## Abstract

**Background:**

Calyceal diverticulum (CD) is a rare congenital anomaly that predisposes patients to stone formation due to impaired urinary drainage. Flexible ureteroscopy with holmium laser incision of the diverticular neck followed by holmium laser lithotripsy for the associated calculi, particularly when combined with a suction-assisted ureteral access sheath, is a favored minimally invasive approach for managing calyceal diverticular stones. While generally safe, vascular complications are exceptionally rare and potentially life-threatening.

**Case presentation:**

We report a rare case involving a 29-year-old man with an upper pole calyceal diverticular stone, who underwent successful fURSL with laser incision of the diverticular neck and complete stone clearance using a suction-assisted access sheath. Five days postoperatively, the patient presented with gross hematuria and flank pain. Conservative management failed, and angiography identified a pseudoaneurysm in an upper-pole branch of the left renal artery. Transcatheter arterial embolization (TAE) was performed, leading to complete resolution of the pseudoaneurysm and bleeding.

**Conclusion:**

This case highlights a rare but serious vascular complication of fURSL in patients with narrow neck calyceal diverticula. Surgeons should be aware of the risks of vascular trauma and consider early angiographic evaluation in the setting of delayed hematuria.

## Introduction

Calyceal diverticulum is a rare congenital anomaly characterized by an outpouching of the renal collecting system into the renal parenchyma, which predisposes patients to urinary stasis and subsequent stone formation (1). Most cases remain asymptomatic and are discovered incidentally during imaging performed for unrelated indications (2). However, the formation of calculi within a diverticulum may lead to clinical symptoms such as flank pain, hematuria, or recurrent urinary tract infections, thereby necessitating urological intervention (3).

Flexible ureteroscopy with holmium:YAG laser lithotripsy (fURSL) has emerged as a favored minimally invasive option for managing calyceal diverticular calculi, alongside percutaneous or laparoscopic approaches that may be considered in selected cases (4, 5). The adjunctive use of suction-assisted ureteral access sheaths can enhance intrarenal visualization and facilitate efficient stone fragment clearance while maintaining low intrarenal pressure (6). In anatomically challenging diverticula, particularly those with a very narrow infundibulum, gaining adequate access may require laser incision of the diverticular neck. Access related maneuvers such as laser incision or dilation may bring the operative field closer to small arterial branches and may introduce a small but potential risk of vascular injury.

Here, we present a rare case involving a young male with a calyceal diverticular stone who underwent flexible ureteroscopy with holmium laser lithotripsy and adjunctive laser incision of the diverticular neck. Although the procedure achieved complete stone clearance, the patient later developed delayed hematuria due to a renal artery pseudoaneurysm, which ultimately required selective transcatheter arterial embolization. This case highlights both the therapeutic success and the potential vascular risks associated with managing stones located within anatomically narrow diverticula.

This case underscores that although suction-assisted fURSL is generally effective and well-tolerated in the treatment of calyceal diverticular stones, clinicians should remain vigilant for rare yet potentially serious vascular complications, including the development of renal artery pseudoaneurysm.

## Case presentation

**Figure 1:**
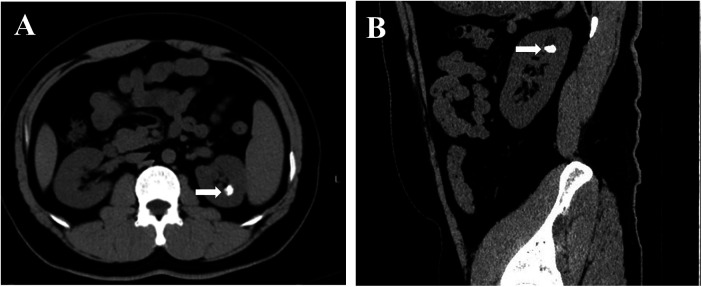
Preoperative CT imaging of calyceal diverticular calculus. **(A)** Coronal non-contrast CT image demonstrating a hyperdense calculus within an upper pole calyceal diverticulum of the left kidney. **(B)** Sagittal CT reconstruction showing the same diverticular stone in the upper-pole region.

A 29-year-old male patient was referred after a renal stone was incidentally detected during his annual routine health examination. A renal ultrasound first revealed a suspicious echogenic focus suggestive of a calculus in the left kidney, which prompted further evaluation. Subsequent non-contrast computed tomography (CT) confirmed a hyperdense calculus measuring approximately 1.5 cm × 1.0 cm located within an upper pole calyceal diverticulum of the left kidney (Figure 1, Supplementary Video 1). Although asymptomatic at the time of diagnosis, the patient elected to proceed with definitive endoscopic management after counseling about the stone characteristics and treatment options. He had no significant past medical or surgical history relevant to stone disease or urological conditions. Preoperative laboratory tests and imaging, including renal function and urine analysis, were unremarkable.

An initial left-sided double-J ureteral stent was placed to facilitate passive ureteral dilation. Two weeks later, the patient underwent flexible ureteroscopy with holmium:YAG laser lithotripsy under general anesthesia. A 0.038-inch hydrophilic guidewire was advanced to the renal pelvis, followed by placement of an 11.5Fr suction-assisted ureteral access sheath to maintain low intrarenal pressure and enhance visibility. Continuous saline irrigation was employed throughout the procedure.

Endoscopic inspection identified a narrow, approximately 2-mm opening of the diverticular neck located dorsally within the upper-pole calyx. To allow entry, the diverticular neck was incised at its visually thinnest portions, approximately at the 10 o'clock and 4 o'clock positions, using a holmium laser (1.0 J, 30 Hz). The incision was extended until the flexible ureteroscope could be advanced smoothly into the diverticulum. Under direct endoscopic visualization, the suction-assisted ureteral access sheath was then guided across the incised neck and gently advanced into the diverticulum, providing access for stone management.

Multiple intradiverticular stones ranging from 5 mm to 1 cm were visualized. Laser lithotripsy was subsequently performed with simultaneous fragment evacuation using the suction-assisted sheath. After confirming complete stone clearance, a 6Fr double-J stent was inserted. The total operative time was approximately 30 min. No active intraoperative bleeding was observed, and the patient was discharged on postoperative day 2 in stable condition.

**Figure 2:**
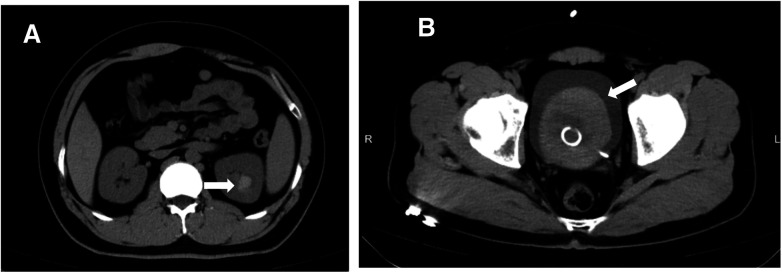
Postoperative CT showing blood clot formation. **(A)** Coronal non-contrast CT image revealing a hyperdense blood clot within the left upper pole calyceal diverticulum. **(B)** Coronal CT image showing a large intravesical blood clot occupying the bladder, with an indwelling ureteral stent visible *in situ*.

On postoperative day 5, the patient presented to the emergency department with acute onset left flank pain and gross hematuria. Non-contrast CT revealed blood clots within both the calyceal diverticulum and urinary bladder (Figure 2, Supplementary Video 2). Initial vital signs were stable, and hemoglobin levels remained within normal limits. Conservative management was initiated, including strict bed rest and continuous bladder irrigation. Intravenous antibiotics were administered empirically due to the presence of gross hematuria and the potential risk of urinary tract infection, although the patient was afebrile at presentation. Serial bladder ultrasonography demonstrated a gradual reduction in intravesical clot burden.

On postoperative day 13, hematuria abruptly worsened following ambulation. Bright red blood was observed in the catheter drainage system, accompanied by intermittent blockage. Manual bladder irrigation yielded large blood clots, and continuous bladder irrigation was resumed. Despite these efforts, the patient experienced persistent hematuria and a progressive decline in hemoglobin over the following 48 h.

**Figure 3:**
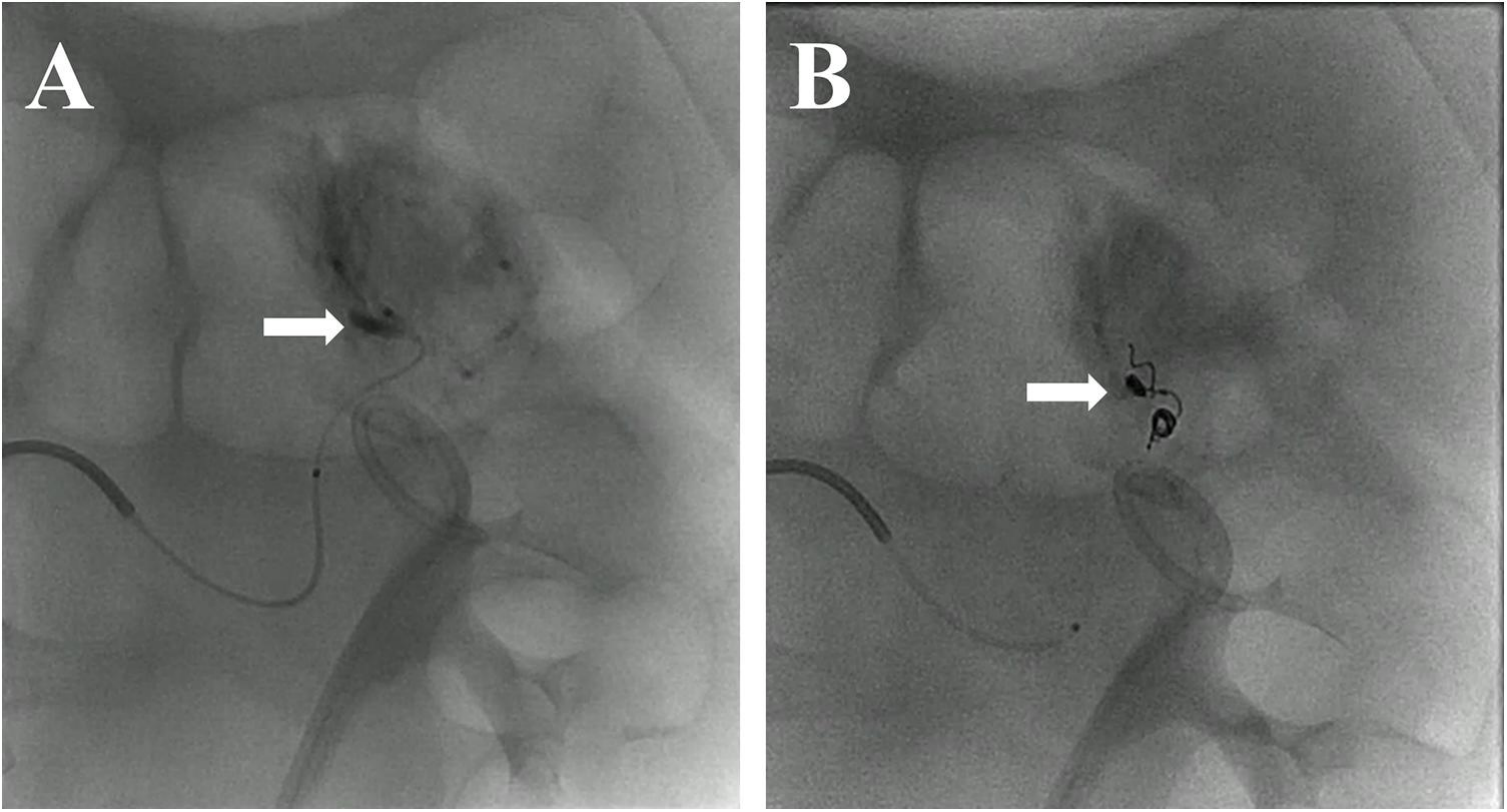
Renal angiography before and after embolization. **(A)** Pre-embolization digital subtraction angiogram (DSA) showing a pseudoaneurysm arising from a branch of the left renal artery. **(B)** Post-embolization image confirming complete obliteration of the pseudoaneurysm, with preservation of the main renal vasculature.

A contrast-enhanced CT with renal vascular phase failed to reveal definitive signs of active extravasation or pseudoaneurysm (Supplementary Video 3). Given continued bleeding, on postoperative day 15, selective digital subtraction angiography was performed and revealed a pseudoaneurysm arising from an upper-pole branch of the left renal artery (Figure 3, Supplementary Video 4). Superselective transcatheter arterial embolization (TAE) was carried out using microcoils, resulting in complete occlusion of the pseudoaneurysm.

Hematuria resolved completely within 24 h of embolization. The patient remained hemodynamically stable and was discharged after one week of observation, with no recurrence of bleeding during a 3-month follow-up. The complete chronological overview of diagnostic procedures, interventions, and clinical outcomes is presented in the timeline (Table 1) for clarity and reference.

**Table 1:** Timeline of clinical events.

Date	Event	Description
January 8, 2025	Initial diagnosis of calyceal diverticular stone	During a routine health check, non-contrast CT revealed a hyperdense calculus measuring approximately 1.5 cm × 1.0 cm within an upper pole calyceal diverticulum of the left kidney. The patient was asymptomatic at that time and chose not to undergo any intervention initially. Surgical treatment was later pursued based on personal preference.
May 7, 2025	Left ureteral stent placement	A left-sided 6Fr double-J stent was placed to facilitate passive ureteral dilation before fURSL. The procedure was uneventful.
May 22, 2025	Flexible ureteroscopy with holmium laser lithotripsy (fURSL)	Stones in the calyceal diverticulum were completely fragmented and removed using a suction-assisted ureteral access sheath. A new 6Fr stent was reinserted. No bleeding observed.
May 27, 2025	Onset of gross hematuria	The patient developed acute flank pain and hematuria on postoperative day 5. CT revealed intrarenal and bladder clots. Conservative management was initiated.
June 6, 2025	Transcatheter arterial embolization (TAE)	Persistent bleeding prompted renal angiography, revealing a pseudoaneurysm in an upper-pole branch. Superselective TAE was performed successfully, resolving hematuria.

## Discussion

CD is often a congenital outpouching of the pelvicalyceal system into the renal parenchyma that may maintain a narrow channel of communication with the collecting system at the calyx or renal pelvis, and is predisposed to stone formation due to urinary stasis. Surgical management is often challenging due to the narrow diverticular neck, which restricts access and visualization. For calculi smaller than 1.5 cm, fURSL is widely regarded as a first-line minimally invasive option (7). However, a narrow diverticular neck often results in limited scope access and therefore necessitates adjunctive measures, such as laser incision of the diverticular neck (3, 8). Once access is achieved, suction-assisted ureteral access sheaths can further improve intrarenal visibility, facilitate efficient fragment evacuation, and help maintain low intrarenal pressure during stone clearance (6). While these methods enhance stone clearance, they may also increase the risk of parenchymal or vascular injury due to thermal or mechanical trauma.

In our case, the most plausible mechanism involves thermal injury from holmium laser incision of a very narrow diverticular neck located within a thin parenchymal area. Delayed renal pseudoaneurysm following laser lithotripsy has been reported in prior studies (9, 10), and the absence of intraoperative bleeding with subsequent delayed hematuria in our patient aligns with a thermally induced mural injury that initially remains sealed but progresses to focal arterial weakening and rupture.

In addition, after the incision, a ureteral access sheath was advanced into the diverticulum under endoscopic guidance to maximize stone clearance. This maneuver likely imposed blunt expansion and traction forces on the incised diverticular neck and its surrounding thin parenchymal tissue. Comparable mechanical stress generated during tract dilation has been suggested as a potential contributor to segmental arterial injury in percutaneous nephrolithotomy (PCNL) (11), and the mechanical dilation performed in our case could plausibly have generated similar stress on adjacent vascular structures.

Renal artery pseudoaneurysm is a recognized but rare complication of endourological procedures, most commonly following PCNL, with an incidence of 0.6%–1.0% (11). In contrast, the occurrence of renal artery pseudoaneurysm following fURSL is exceedingly uncommon, and previously reported cases have typically been associated with prolonged operative time, excessive laser energy use, or procedures performed in anatomically complex kidneys such as malrotated or duplex systems (9, 12–15).

To the best of our knowledge, no prior report has described delayed hemorrhage secondary to holmium laser incision of a calyceal diverticular neck in combination with the use of a suction-assisted ureteral access sheath. Although suction-assisted access sheaths themselves are not known to directly cause pseudoaneurysm, the combination of laser incision and subsequent mechanical dilation within a thin parenchymal zone may have contributed to delayed arterial wall disruption and pseudoaneurysm formation. This highlights a potential combined mechanism of vascular trauma, suggesting that both laser incision within a thin parenchymal zone and subsequent advancement of a suction-assisted access sheath should be performed with caution in diverticular stones with narrow infundibula.

The diagnosis of pseudoaneurysm requires a high index of suspicion, particularly in patients presenting with delayed hematuria following fURSL. While contrast-enhanced CT is a useful non-invasive tool, it may fail to detect small or intermittently bleeding lesions, as in our patient. In such cases, selective renal angiography remains the gold standard for both diagnosis and treatment. TAE is safe, minimally invasive, and offers definitive management with preservation of renal parenchyma and function.

## Patient perspective

The patient initially decided against treatment because he was asymptomatic and did not consider the stone problematic. After learning more about the condition, he later chose to undergo surgery. Following the unexpected bleeding, he expressed surprise and some dissatisfaction that such a minimally invasive procedure could lead to a serious complication. Nevertheless, he appreciated the rapid response of the medical team and was satisfied with his full recovery and preserved kidney function.

## Conclusions

Flexible ureteroscopy with holmium laser lithotripsy remains a safe and effective treatment for calyceal diverticular calculi. Nevertheless, surgeons should exercise caution in anatomically narrow diverticula, where excessive laser or suction use may cause vascular trauma. Early recognition and timely angiographic management are essential for favorable outcomes.

## Data Availability

The original contributions presented in the study are included in the article/Supplementary Material, further inquiries can be directed to the corresponding author.

## Supplementary material

Supplementary Video 1 Preoperative non-contrast CT scan showing a hyperdense calculus within the left upper pole calyceal diverticulum.

Supplementary Video 2 Postoperative day 5 (May 27, 2025) non-contrast CT scan demonstrating hyperdense blood clots within the calyceal diverticulum and urinary bladder.

Supplementary Video 3 Postoperative day 13 (June 4, 2025) contrast-enhanced CT scan performed during the evaluation of persistent hematuria, showing no obvious extravasation or vascular abnormality.

Supplementary Video 4 Postoperative day 15 (June 6, 2025) digital subtraction angiography (DSA) revealing a pseudoaneurysm arising from an upper-pole branch of the left renal artery, with successful superselective embolization.

## References

[1] Waingankar N Hayek S Smith AD Okeke Z. Calyceal diverticula: a comprehensive review. Rev Urol. (2014) 16(1):29–43. 24791153 PMC4004282

[2] Hulbert JC Reddy PK Hunter DW Castaneda-Zuniga W Amplatz K Lange PH. Percutaneous techniques for the management of caliceal diverticula containing calculi. J Urol. (1986) 135(2):225–7. 10.1016/S0022-5347(17)45590-X 3080605

[3] Novăcescu D Dema V Croitor A Laţcu S. Flexible ureterorenoscopy: practical considerations and a glimpse to the future-A narrative review. Rom J Urol. (2024) 23(1):19–30.

[4] Gonzalez RD Whiting B Canales BK. Laparoscopic calyceal diverticulectomy: video review of techniques and outcomes. J Endourol. (2011) 25(10):1591–5. 10.1089/end.2011.0163 21830911

[5] Pan Y Chen G Chen H Zhu Y Chen H. The left ureterocele and stone of calyceal diverticulum in the patient with bilateral incomplete duplex kidneys managed by flexible ureteroscopy: a case report and literature review. BMC Urol. (2020) 20(1):35. 10.1186/s12894-020-00604-7 32228555 PMC7106577

[6] Chen H Xiao J Ge J Liu T. Clinical efficacy analysis of tip-flexible suctioning ureteral access sheath combined with disposable flexible ureteroscope to treat 2–4 cm renal stones. Int Urol Nephrol. (2024) 56(10):3193–9. 10.1007/s11255-024-04072-y 38717576 PMC11405463

[7] Zeng SP Sun YF Yu HY Yang J Deng KF. Efficacy of flexible ureterorenoscopy with holmium laser in the management of calyceal diverticular calculi. Urolithiasis. (2024) 52(1):50. 10.1007/s00240-024-01552-9 38554174 PMC10981604

[8] Soloway M. Urinary tract infection and a small stone. Curr Urol Rep. (2007) 8(4):255–8. 10.1007/s11934-007-0069-6 18519008

[9] Yin C Chen F Jiang J Xu J Shi B. Renal pseudoaneurysm after holmium laser lithotripsy with flexible ureteroscopy: an unusual case report and literature review. J Int Med Res. (2023) 51(3):3000605231162784. 10.1177/03000605231162784 36974990 PMC10052483

[10] Deng XX Zhang W Fu D Fu B. Renal pseudoaneurysms after flexible ureteroscopy and holmium Laser lithotripsy: a case report. Front Surg. (2022) 9:896548. 10.3389/fsurg.2022.896548 36034371 PMC9406514

[11] Shah S Fatima A Shah MDA Ali W Gorya IA Nasrullah F. Post-PCNL renal artery pseudoanurysm. J Coll Physicians Surg Pak. (2018) 28(3):238–9. 10.29271/jcpsp.2018.03.238 29544585

[12] Al Barajraji M Coscarella M Holz S Moussa I Naudin M Brassart N. Occult renal artery pseudoaneurysm causing persistent hematuria after flexible Thulium fibered laser lithotripsy: a case report and literature review of rare but potentially fatal complication. Radiol Case Rep. (2023) 18(10):3525–8. 10.1016/j.radcr.2023.07.030 37547792 PMC10400803

[13] Asi T Khamashta N Dalal A. Renal artery pseudoaneurysm presenting in a single functioning kidney with prior partial nephrectomy following flexible ureterorenoscopy: a case report. J Surg Case Rep. (2024) 2024(9):rjae594. 10.1093/jscr/rjae594 39291252 PMC11407829

[14] Corrales M Hasan MN Carioti GE Emiliani E Doizi S Traxer O. Arterial pseudoaneurysm: a rare complication following laser lithotripsy-case series and literature review. World J Urol. (2024) 42(1):280. 10.1007/s00345-024-04980-9 38693433

[15] Gauhar V Traxer O Woo SJQ Fong KY Ragoori D Wani A PCNL vs RIRS in management of stones in calyceal diverticulum: outcomes from a global multicentre match paired study that reflects real world practice. World J Urol. (2023) 41(11):2897–904. 10.1007/s00345-023-04650-2 37864647

